# Evaluation of the cobas plasma separation card to identify HIV-infected patients in virological failure in real-life conditions in Vietnam

**DOI:** 10.1371/journal.pone.0329841

**Published:** 2025-09-17

**Authors:** Hanh Thi Hong Ngo, Binh Thanh Nguyen, Tram Thi Phuong Pham, Nhung Thi Hong Le, Hien Ba Pham, Trang Thi Thu Nguyen, Mohand Ait-Ahmed, Tuan Anh Nguyen, Thang Hong Pham, Yoann Madec

**Affiliations:** 1 National Reference Laboratory of HIV Molecular Biology, National Institute of Hygiene and Epidemiology, Hanoi, Vietnam; 2 Dong Da General Hospital, Hanoi, Vietnam; 3 Nam Tu Liem District Medical Center, Hanoi, Vietnam; 4 Center for Translational Research, Institut Pasteur, Université de Paris, Paris, France; 5 Epidemiology of Emerging Diseases Unit, Institut Pasteur, Université de Paris, Paris, France; Kimura Hospital, JAPAN

## Abstract

**Background:**

The standard of care for HIV-infected patients on antiretroviral therapy (ART) relies on regular monitoring of HIV viral load. Access to viral load monitoring has improved in recent years, but remains scarce in some settings. Alternatives to plasma, such as dried specimens, can help bring routine viral load testing to remote settings. This study aimed to evaluate performances of the cobas® plasma separation card (PSC) to detect virological failure at the threshold of 1,000 copies/mL in real-life conditions in Vietnam.

**Setting:**

Patients were enrolled in two hospitals in Hanoi (Vietnam).

**Methods:**

This cross-sectional evaluation enrolled 250 HIV-infected adults using convenience sampling. All provided plasma and PSC samples. The sensitivity and specificity of PSC, as compared to plasma, to identify patients in virological failure were estimated. Convenience sampling was used to reach the target numbers of 150 and 100 patients with plasma viral load ≥ and <1,000 copies/mL, respectively.

**Results:**

Overall, 250 patients were enrolled. Of the 127 (50.8%) patients with plasma viral load ≥1,000 copies/mL, the PSC viral load was also ≥1,000 copies/mL in 126 (sensitivity (95% confidence interval (CI)): 99.2% (95.7–99.9)). Of the 123 (49.2%) patients with plasma viral load <1,000 copies/mL, the PSC viral load was also <1,000 copies/mL in 108 (specificity (95% CI): 87.8% (80.7–93.0)). Overall, the concordance rate was 93.6%.

**Conclusions:**

This field evaluation of the cobas® PSC showed its high effectiveness in identifying patients in virological failure. The specificity was 87.8%, lower than in previous reports. However, in cases of discrepancy, the PSC viral load level was never far from the 1,000 copies/mL threshold, which may be due to measurement error inherent to the technique.

## Introduction

Following World Health Organization (WHO) recommendations, the standard of care for HIV-infected patients on antiretroviral therapy (ART) relies on regular monitoring of HIV viral load (VL) [1]. HIV VL monitoring is essential to detect suboptimal adherence or virological failure, and in the latter case to adapt the ART combination. In low-and middle-income countries, national guidelines for HIV care essentially follow WHO recommendations [1].

UNAIDS launched its 95-95-95 targets to end the AIDS epidemic by 2025 [2]. Following these targets, in 2025, 95% of people living with HIV should know their HIV status, of whom 95% should be on ART, and of the latter 95% should be virologically suppressed. In 2021, UNAIDS estimated that 92% of those on ART were virologically suppressed [3], close to the target. However, tremendous disparities between countries persist [3].

To increase the number of HIV-infected patients on ART, the need for VL monitoring is growing. Access to VL monitoring has progressed but remains complex in some settings. Plasma is the preferred sample type for HIV VL testing and is considered the gold standard. Low access to VL monitoring in certain areas is jointly explained by their remoteness and the difficulty in conveying plasma samples to a laboratory able to measure HIV VL, as plasma must be shipped with a strict cold chain to prevent nucleic acid deterioration [4,5]. In recent years, point-of-care (POC) devices have been developed. Some of them, showing very good performances compared to plasma [6], can help improve HIV VL monitoring coverage. Yet POC devices still necessitate a well-organized supply circuit and can be costly if the volume of testing at the care site is not high enough [7]. Blood sampling using dried blood spots (DBS) is another option to improve HIV VL monitoring coverage, as mentioned in the WHO guidelines [1]. If DBS are an appealing tool as they do not require new machines, special attention must be paid to the choice of technique as their performances are not all equivalent [6,8].

A disadvantage of DBS lies in the risk of proviral DNA amplification, which could lead to overestimation of the HIV VL level [9], explaining a lower specificity. To overcome this defect, some researchers have suggested collecting dried plasma spots (DPS) with good but inconsistent overall results [10]. Moreover, the collection of DPS, as opposed to DBS, requires centrifugation, which is out of reach for some laboratories.

Recently, paper cards have been developed, making it possible to deposit whole blood but separate plasma from the remaining blood components through layers of filter papers. The cobas® plasma separation card (PSC) has shown very good performances in both sensitivity and specificity to detect virological failure at the threshold of 1,000 copies/mL [11–13].

In this study, we aimed to evaluate the sensitivity and specificity of the cobas® PSC to detect virological failure at the threshold of 1,000 copies/mL in real-life conditions in Vietnam.

## Methods

The study was a cross-sectional evaluation enrolling HIV-infected adults (≥18 years) who visited Nam Tu Liem and Dong Da outpatient clinics (OPCs) in Hanoi (Vietnam) for routine care between September 7, 2022 and February 15, 2023.

The main goal of the study was to estimate the sensitivity and specificity of the cobas® PSC, as compared to plasma, to identify patients in virological failure defined at 1,000 copies/mL [1], the current threshold used in Vietnam.

Under the assumption that sensitivity is 98%, for the lower limit of the 95% confidence interval not to be below 94%, the enrollment of 150 patients with plasma VL ≥ 1,000 copies/mL was targeted. Based on prior experience, specificity (i.e., the ability to identify patients with VL < 1,000 copies/mL) was expected to be less of an issue [14]. Therefore, under the assumption that specificity is 98%, for the lower limit of the 95% confidence interval not to be below 93%, the enrollment of 100 patients with plasma VL < 1,000 copies/mL was targeted. Overall, the target number of patients to be enrolled was 250. We used convenience sampling to meet our enrollment targets. Given the high rate of virological success usually documented in Vietnam [15–17], to find plasma VL ≥ 1,000 copies/mL, we sought patients not yet on ART or on ART for <3 months.

### Blood sampling and sample preparation

At the OPC laboratory, 6 mL of venous blood were drawn in EDTA tubes. Immediately after collection, the blood sample was stored in a cool box. Within three hours, all samples were transferred to the HIV molecular laboratory at the National Institute of Hygiene and Epidemiology (NIHE) in Hanoi (Vietnam).

Upon reception at the HIV molecular laboratory, 140 µL of whole blood was spotted on each of the three spots of two cobas® PSC using calibrated pipettes. The PSC were left to dry at ambient temperature, directly on the workbench, for a minimum of three hours. After drying, the PSC were placed individually in Ziploc bags with 4 g of desiccants (i.e., 4 bags) and kept at ambient temperature (monitored daily) in a dedicated box for seven days before being tested, then stored at −80°C if the testing was incomplete.

The remaining whole blood was centrifugated for 20 minutes at 2,500 rpm, after which plasma was collected in 2-mL cryotubes. Plasma samples were stored immediately at −80°C unless they were to be tested within the following 24 hours.

### HIV VL testing

All the plasma and PSC samples were processed for VL testing using the cobas® HIV-1 quantitative nucleic acid test on the cobas® 4800 System (Roche, Basel, Switzerland), according to the manufacturer’s instructions. The cobas® 4800 System consists of two separate devices, the cobas® × 480 for sample preparation and cobas® z 480 analyzer for amplification/detection. Real-time detection and discrimination of PCR products are completed by measuring the fluorescence of the released reporter dyes for the viral targets and RNA QS, respectively.

Plasma samples (1,000 µL) were loaded in the cobas® 4800 System, 400 µL was the sample processing volume, and the lower limit of detection [LoD] was established at 20 copies/mL.

For PSC samples, the top layers were removed to access the DPS. A single pre-cut DPS was taken from the card using forceps to extract the spot, the forceps needing sterilization after each use (dip in the sodium hypochlorite solution 10% for one minute and transfer to the isopropanol 70% for one minute to disinfect). The retrieved DPS was incubated at 56°C with 800 μL of Specimen Pre-Extraction Reagent (SPER-Roche) for 10 minutes at 1,000 rpm on a preheated thermomixer. Samples were then loaded in the cobas® 4800 System without removing the DPS for automatically processing 400 µL with the LoD at 599 copies/mL [18].

Plasma and PSC samples (after incubation) have the same analytical workflow and processing volume on the cobas® 4800 System. VL results were interpreted according to the manufacturer’s instructions. Of note, the same two technicians throughout the study handled the preparation and testing of all samples.

### Statistical analysis

In patients for whom both plasma and PSC VL levels were above the corresponding lower detection limit, the concordance between VL measurements on plasma and on PSC was investigated using a Bland–Altman analysis.

The sensitivity and specificity of the PSC and their corresponding confidence intervals (CI), as compared to plasma, to identify patients in virological failure at the threshold of 1,000 copies/mL were estimated. Sensitivity was the proportion of patients with VL ≥ 1,000 copies/mL on PSC among those who presented with a plasma VL ≥ 1,000 copies/mL. Specificity was the proportion of patients with VL < 1,000 copies/mL on PSC among those who presented with a plasma VL < 1,000 copies/mL.

To investigate if some conditions (e.g., freezing PSC prior to VL testing) influenced the performances of PSC, stratified analyses were conducted, estimating the sensitivity and specificity in each category. The sensitivity (and specificity) was then compared between categories using a chi-2 test.

A post-hoc analysis was conducted in 23 randomly selected PSC samples to investigate reproducibility.

All analyses were performed using Stata 17 (Stata Corps., College Station, TX, USA).

### Ethics approval and consent to participate

The study was approved by the institutional review boards at the Institut Pasteur in Paris (France) and at the NIHE in Hanoi (Vietnam). All methods were performed in accordance with guidelines and regulations relevant to human research. Written informed consent was obtained from all participants.

## Results

Overall, 250 HIV-infected patients were enrolled: 170 (68.0%) in Nam Tu Liem OPC and 80 (32.0%) in Dong Da OPC. Of these patients, 199 (79.6%) were male, and the median (interquartile range (IQR)) age was 34 (26–44) years.

Plasma VL testing failed in five (2.0%) samples out of 250 due to clotting, but they were retested successfully in the subsequent batch. Plasma VL was measured in median (IQR) 8 (2–17) days after blood collection. Plasma VL was below the lower LoD in 64 (25.6%) patients, was detected but <1,000 copies/mL in 59 (23.6%) patients and was ≥ 1,000 copies/mL in the remaining 127 (50.8%) patients. In the 186 plasma samples above the lower LoD, median (IQR) plasma viral load was 4.19 [2.67–5.08] log copies/mL (Table 1).

**Table 1:** Description of viral load measurements (N = 250).

	Plasma	PSC	Plasma and PSC
N (%)> lower limit of detection	186 (74.4)	152 (60.8)	150 (60.0)
N (%) ≥1,000 copies/mL	127 (50.8)	141 (56.4)	126 (50.4)
Median (IQR) in log copies/mL*	4.19 (2.67–5.08)	4.69 (3.80–5.42)	–
Median (IQR) difference in log copies/mL**	–	–	+0.23 (0.04–0.45)

PSC: plasma separation card; IQR: interquartile range

*Only considering those with viral load > lower limit of detection; **difference is PSC viral load minus plasma viral load

In 245 (98.0%) patients, PSC were created on the same day as the whole blood sample was collected and arrived at the HIV molecular laboratory at the NIHE. In the remaining five (2.0%) patients, PSC were created on the following day. PSC VL testing failed in 10 (4.0%) of the 250 samples, but they were retested successfully in the subsequent batch. PSC VL was measured in median (IQR) 20 (14–29) days after creation. PSC VL was below the lower LoD in 98 (39.2%) patients, was detected but <1,000 copies/mL in 11 (4.4%) patients and was ≥ 1,000 copies/mL in the remaining 141 (56.4%) patients. In the 152 PSC samples above the lower LoD, median (IQR) PSC viral load was 4.69 [3.80–5.42] log copies/mL (Table 1).

### Quantitative comparison of PSC versus plasma

**Fig 1:**
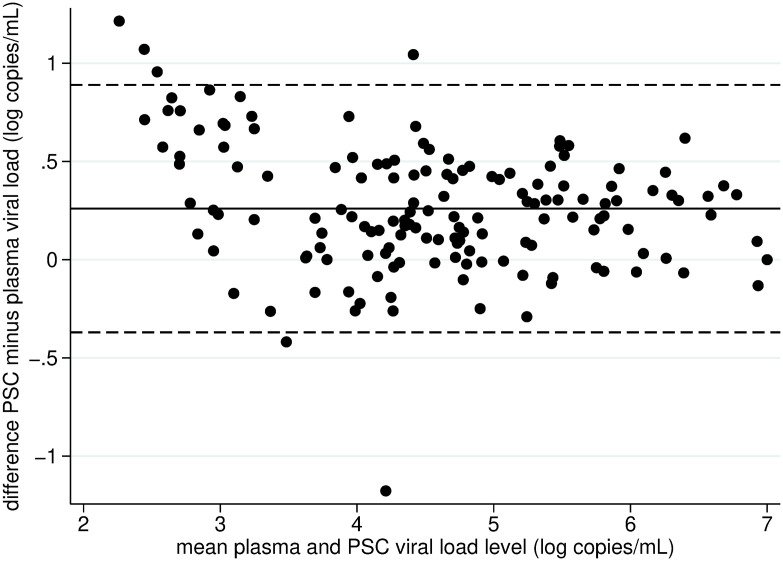
Bland–Altman analysis to evaluate the concordance between plasma and PSC viral load levels in 150 patients with both plasma and PSC viral load measurements above the lower limits of detection (the full line represents the mean difference between plasma and PSC viral load measurements (+0.26 copies/mL); the dashed lines represent the 95% confidence interval of the difference between plasma and PSC viral load measurements). PSC: plasma separation card.

In 150 patients, both plasma and PSC VL measurements were above their respective lower LoD. The mean difference was 0.26 log copies/mL (Table 1), indicating higher values in PSC than in plasma; in 28 (18.7%) cases, however, the VL level was lower in PSC than in plasma. The difference was larger than 0.5 log copies/mL in 31 (20.7%) cases. The Bland–Altman analysis (Figure 1) also shows a tendency towards higher levels in PSC than in plasma, but the difference was stable whatever the VL level.

### Qualitative comparison of PSC versus plasma

Of the 127 patients with plasma VL ≥ 1,000 copies/mL, the PSC VL was also ≥1,000 copies/mL in 126, corresponding to a sensitivity (95% CI) of 99.2% (95.7–99.9) (Table 2). The only patient with downward misclassification showed a plasma VL of 1,410 copies/mL while the PSC VL was < 599 copies/mL. Of the 123 patients with a plasma VL < 1,000 copies/mL, the PSC VL was also <1,000 copies/mL in 108, corresponding to a specificity (95% CI) of 87.8% (80.7–93.0). Overall, the concordance rate was 93.6%.

**Table 2:** Sensitivity and specificity of the cobas® plasma separation card (PSC), as compared to plasma, at the threshold of 1,000 copies/mL.

	Plasma viral load ≥1,000 copies/mL	Plasma viral load <1,000 copies/mL	Total
PSC viral load ≥1,000 copies/mL	126 (sensitivity: 99.2%)	15	141
PSC viral load <1,000 copies/mL	1	108 (specificity: 87.8%)	109
Total	127	123	250

PSC: plasma separation card

Of the 15 patients with upward misclassification, one presented with an undetectable plasma VL while the PSC VL was 1,070 copies/mL. In the 14 other patients, the plasma VL ranged from 115 copies/mL to 813 copies/mL, while the PSC VL ranged from 1,040 copies/mL to 3,940 copies/mL. In these 14 patients, the median (IQR) difference was 0.7 (0.6–0.8) log copies/mL, and was < 0.5 log copies/mL in three patients. If these three patients were considered as correctly classified, the specificity (95% CI) would be 90.2% (83.6–94.9).

Some PSC were frozen prior to VL testing. To investigate whether freezing could impact the performances, sensitivity and specificity were estimated if VL was tested on PSC that were not frozen and had been previously frozen. Neither sensitivity nor specificity differed between the two conditions (p = 0.99 and p = 0.85, respectively; Table 3). Prior to freezing PSC were kept at a mean ambient temperature of 21.1°C. When viral load was measured on frozen PSC, the PSC had been kept frozen for a median (IQR) of 13 (7–18) days.

**Table 3:** Effect of freezing and duration from plasma separation card (PSC) collection to viral load testing on the sensitivity and specificity.

	Plasma VL ≥ 1,000 copies/mL	Sensitivity (95% CI)	p-value	Plasma VL < 1,000 copies/mL	Specificity (95% CI)	p-value
PSC freezing			0.99			0.84
No	60	100 (94.0–100)		52	88.5 (76.6–95.6)	
Yes	67	98.5 (92.0–99.9)		71	87.3 (77.3–94.0)	
Duration			0.36			0.70
<21 days	81	100 (95.5–100)		55	89.1 (77.8–95.9)	
≥21 days	46	97.8 (88.5–99.9)		68	86.8 (76.4–93.8)	

VL: viral load; CI: confidence interval; PSC: plasma separation card

The PSC conservation duration prior to VL measurement was also investigated by defining two categories based on the median duration: PSC VL measured <21 days or ≥21 days from PSC collection. Neither sensitivity nor specificity differed by PSC conservation time (p = 0.36 and p = 0.70, respectively; Table 3).

Forceps were used to extract the DPS from the card, the forceps needing sterilization after each use. To investigate if cross-contamination could explain the 15 discrepancies observed, we looked to see if the VL measured in the well preceding each discrepancy was ≥ 1,000 copies/mL or not. This was the case in half of the discrepancies (8/15), while the level was lower in the previous well in the remaining cases.

Finally, to have an idea of the reproducibility of the PSC VL measurements, we randomly selected 23 samples tested on January 11, 2023, when only one incubation batch was needed, and remeasured the PSC VL level. The median (IQR) difference between the two PSC VL results was −0.01 (−0.11 to +0.08) and ranged from −0.30 to +0.19 log copies/mL (Table 4). In two cases, the difference was not estimated as at least one of the two VL results was undetected.

**Table 4:** Reproducibility of the plasma separation card (PSC) viral load measurement on 23 randomly selected PSC samples.

1^st^ PSC viral load measure (copies/mL)	2^nd^ PSC viral load measure (copies/mL)	Difference (in log copies/mL)
<599	<599	–
796	<599	–
2,240	1,120	−0.30
6,040	4,740	−0.11
7,190	11,200	+0.19
8,140	9,950	+0.03
12,300	13,300	+0.02
17,400	14,100	−0.09
17,800	19,000	+0.03
24,200	22,600	−0.03
44,200	33,200	−0.12
53,200	64,000	+0.08
59,700	91,500	+0.19
63,100	55,700	−0.05
149,000	150,000	+0.01
218,000	241,000	+0.04
230,000	256,000	+0.05
247,000	204,000	−0.08
342,000	198,000	−0.24
597,000	893,000	+0.17
1,290,000	918,000	−0.15
3,180,000	2,450,000	−0.11
9,400,000	7,800,000	−0.08

## Discussion

This field evaluation of the cobas® PSC in routine conditions showed its high effectiveness in identifying patients in virological failure at the threshold of 1,000 copies/mL, with a sensitivity (95% CI) of 99.2% (95.7–99.9). Our result confirms those previously observed in other settings [11–13,19]. A recent evaluation in real-life settings found a lower sensitivity of 87.5%, which the authors qualified as acceptable [20]. Lower sensitivity is, however, an issue as patients would be wrongly identified as in virological success and maintained on a failing ART regimen.

On the other hand, our identification of patients with a VL < 1,000 copies/mL was deceiving as the specificity (95% CI) was only 87.8% (80.7–93.0), much lower than the first evaluations published [11–13,20], but similar to a more recent one [19]. This result was due to an overestimation of VL in PSC as compared to plasma. When three discordant samples that presented a difference <0.5 log copies/mL were considered as concordant, the specificity (95% CI) was improved to 90.2% (83.6–94.9), albeit without reaching the level of previous evaluations.

Overall, correct classification occurred in 93.6% of the samples, and the PSC’s performances compared well with those reported by four other technologies that were qualified as acceptable for HIV VL monitoring in a recent meta-analysis [8].

Nevertheless, one can argue that poor specificity is less of an issue than poor sensitivity. Indeed, in cases of VL above the 1,000 copies/mL threshold, the patient would undergo adherence support sessions and then VL would be retested within three to four months. Given the sensitivity observed, it appears statistically unlikely that a true VL below the threshold would again be above the 1,000 copies/mL threshold when remeasured. Nevertheless, these unnecessary VL measurements represent a cost burden for national programs. Moreover, receiving a VL result suggestive of therapeutic failure may induce stress and anxiety in the patient. Hopefully, counselling and adherence support can help the patient maintain high adherence or even improve it. It is also important to note that in the fifteen patients with upward misclassification on PSC, the plasma VL was detectable in all but one, suggesting that residual low-level viral replication was ongoing in these patients, and that enhanced adherence to ART could benefit them.

Yet we wondered what could explain the lower specificity in our study. Some samples were tested after freezing, and we investigated whether this could have impacted the specificity. However, the specificity was not significantly different when PSC were tested after remaining at room temperature only or after freezing at −20°C. The duration of PSC conservation did not impact the specificity either.

PSC sample preparation for HIV VL testing required us to remove the top layers of the card to reach the layer where plasma migrated. The spot was then extracted by sterile forceps. After each sample, the forceps must be sanitized before being reused. This means extra manipulation for the laboratory staff, increasing the risk of cross-contamination if the forceps were not correctly sanitized. We used an empirical approach to investigate if cross-contamination could explain the specificity observed in our study by looking at the VL level in the well preceding the well where a discrepancy was identified. In half of the cases, the PSC VL in the previous well was indeed higher than 1,000 copies/mL, but in the remaining half it was lower than 1,000 copies/mL. Although cross-contamination could not formally be ruled out, we believe that it is highly unlikely to explain our results: the laboratory staff is very experienced and standard operating procedures were implemented.

In cases of discrepancy, the PSC VL level was within a reasonable distance from the 1,000 copies/mL threshold and was always <4,000 copies/mL, in line with a previous evaluation [13]. It is possible that these discrepancies are explained by measurement error inherent to the technique, as it has been reported that variability is larger at lower VL levels [11,13]. Although the PSC is supposed to provide a plasma matrix free of cell-associated RNA, contamination could occur when whole blood is spotted, e.g., if the quantity did not respect requirements.

This evaluation was conducted on the cobas® 4800 instrument, while previous evaluations involved the cobas® 6800/8800 instrument [11,13]. Although the manufacturer mentions PSC use in the instrument specifications, PSC preparation is different in the cobas® 4800 than with other cobas®. When using PSC samples, the cobas® 4800 processed a volume of 400 μL while the other cobas® processed 850 μL. As illustrated on HCV VL testing, although the correlation was good, the cobas® 4800 system led to lower HCV VL levels than when using cobas® 6800 [21]. Perhaps use of the cobas® 4800 system explains the lower specificity we observed for identifying HIV virological failure at the 1,000 copies/mL threshold, potentially due to conversion factors or to intrinsic assay performance. Additional studies using the cobas® 4800 system could help clarify this issue.

From a practical standpoint, of the 250 PSC samples tested, failure occurred in 10 samples and 1 even had to be tested 3 times before succeeding. Each time, the error message suggested the volume automatically extracted was too low for VL testing, likely due to the paper spot left in the tube loaded in the instrument. As previously mentioned, preparation of the PSC samples requires the use of sterile forceps to extract the dried plasma spot. This step can raise the risk of cross-contamination if the forceps are not correctly sanitized or if the laboratory is not well organized. Although plasma must be vortexed and spun before VL testing, PSC preparation remains more time-consuming for the laboratory staff as it requires several extra steps.

In terms of quantitative difference, PSC VL was overestimated as compared to plasma. The mean difference, estimated when results were above the detection limit in both sample types, was 0.23 log copies/mL higher on PSC than on plasma. The mean difference is higher than reported in other studies [11]. Nevertheless, PSC showed good reproducibility on the 24 PSC samples tested twice, as all absolute differences were <0.3 log copies/mL.

One strength of this study was its conduct in field conditions in terms of PSC conservation, in a laboratory performing routine VL testing for the northern provinces of Vietnam. Sample preparation, for both plasma and PSC, was however centralized at the laboratory performing VL testing to optimize sample quality. Nevertheless, previous studies have shown that blood spotting is not an issue at clinical site level, even if calibrated blood quantities must be used. Another limitation was the use of convenience sampling, which does not reflect the prevalence of virological failure, and therefore did not make it possible to estimate the positive and negative predictive values.

## Conclusions

The PSC performances found in our study, and confirmed in other settings, were not as high as initial evaluations had us expect. With a high sensitivity, PSC showed near-perfect identification of patients truly in virological failure. The specificity showed an acceptable ability to detect those in virological success. Indeed, the cobas® PSC led to some upward misclassification; however, in cases of VL above the threshold defining failure, VL would be remeasured after adherence support, which would likely confirm the real virological status. PSC qualifies to join the arsenal already available to improve VL coverage, which remains too low in some settings.
